# Many Mg–Mg bonds form the core of the Mg_16_Cp*8Br_4_K cluster anion: the key to a reassessment of the Grignard reagent (GR) formation process?†
†Electronic supplementary information (ESI) available: Materials and methods, supplementary text. See DOI: 10.1039/c5sc03914b



**DOI:** 10.1039/c5sc03914b

**Published:** 2015-11-26

**Authors:** T. Kruczyński, F. Henke, M. Neumaier, K. H. Bowen, H. Schnöckel

**Affiliations:** a Karlsruhe Institute of Technology (KIT) , Institute of Inorganic Chemistry , Engesserstr. 15 , D-76131 Karlsruhe , Germany . Email: hansgeorg.schnoeckel@kit.edu ; Fax: +49 721 608 44854; b Karlsruhe Institute of Technology (KIT) , Institute of Meteorology and Climate Research - Atmospheric Trace Gases and Remote Sensing , H.-v.-Helmholtz-Platz 1 , 76344 Leopoldshafen , Germany; c Johns Hopkins University , Department of Chemistry , 3400 North Charles St. , Baltimore , MD 21218 , USA

## Abstract

It caused a sensation eight years ago, when the first room temperature stable molecular compound with a Mg–Mg bond (LMgMgL, L = chelating ligand) containing magnesium in the oxidation state +1 was prepared. Here, we report the preparation of a [Mg_16_Cp*8Br_4_K]^–^ cluster anion (Cp* = pentamethylcyclopentadiene) with 27 Mg–Mg bonds. It has been obtained through the reaction of KCp* with a metastable solution of MgBr in toluene. A highly-resolved Fourier transform mass spectrum (FT-MS) of this cluster anion, brought into vacuum by electrospraying its solution in THF, provides the title cluster's stoichiometry. This Mg_16_ cluster together with experiments on the metastable solution of MgBr show that: during the formation process of GRs (Grignard reagents) which are involved in most of sophisticated syntheses of organic products, not the highly reactive MgBr radical as often presumed, but instead the metalloid Mg_16_Cp*8Br_4_ cluster anion and its related cousins that are the operative intermediates along the pathway from Mg metal to GRs (*e.g.* Cp*MgBr).

## Abstract 2

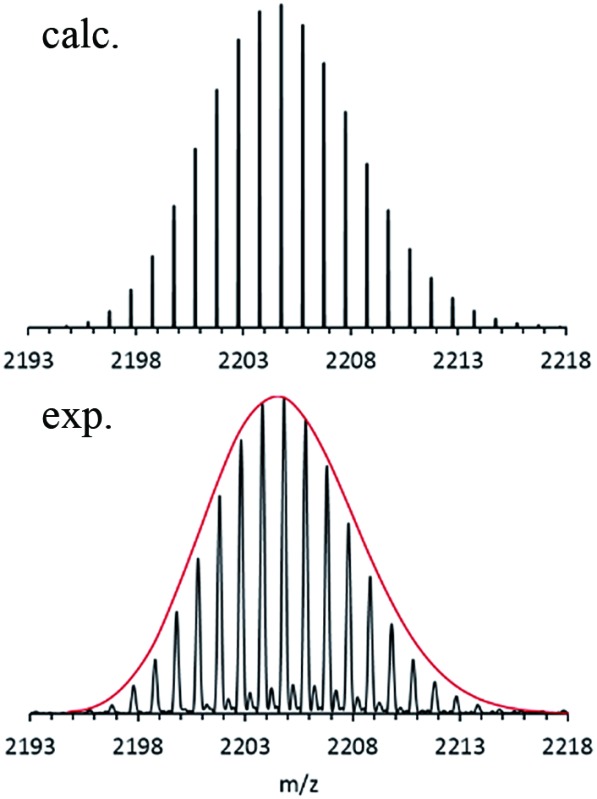
It caused a sensation eight years ago, when the first room temperature stable molecular compound with a Mg–Mg bond (LMgMgL, L = chelating ligand) containing magnesium in the oxidation state +1 was prepared.

## Introduction

Since the preparation of the first two molecular compounds, LMg-MgL (L = chelating ligand) containing a Mg–Mg σ-bond in 2007,^1^ other examples incorporating different β-diketiminate, diimine-enolate and α-diimine ligands (including some adducts) have been reported by Jones^2^ and Wu.^3^ The reactivity of these reagents has also been investigated intensively.^4–8^ In all these compounds, one strong Mg–Mg bond with a bond energy of about 200 kJ mol^–1^ is present, this having been determined from the resonance Raman spectra of matrix isolated Mg_2_Cl_2_.^9^ Since the chelating ligands also form strong and protective MgL bonds, these Mg(i) compounds are kinetically stable against heating, and thus the thermodynamically favored disproportionation to Mg metal and Mg(ii) moieties is avoided. Therefore, no molecular intermediates formed during heating and decomposition of LMgMgL molecules could be trapped on their way to the Mg metal, *i.e.* no metalloid‡
‡Suffix-*oid* comes from the greek *ειδος* (ideal, prototype): The ideal geometry of a metal structure is visible in the cluster. A more detail definition of these clusters was introduced by us 15 years ago (*cf.* ESI†). clusters (Mg_*n*_R_*m*_, *n* > *m*) containing many Mg–Mg bonds were obtained.

In order to trap, for the first time, metalloid magnesium clusters as intermediates between magnesium and magnesium(ii) species [either in the gas phase, *e.g.* between naked Mg_*n*_ clusters^10^ and oligomeric MgX_2_ (X = halide) molecules, or in the solid state], we developed a novel synthesis of a metastable MgBr solution.^11^ This procedure was in principle similar to the experimentally sophisticated synthesis of metastable solutions of the monohalides of group 3 elements (*e.g.* AlCl, GaCl), from which, *via* their disproportionation and substitution reaction, metal-rich intermediates have been obtained.^12,13^ These so called metalloid clusters (defined as clusters containing more metal–metal than metal ligand bonds), which were described as a curiosity twenty years ago,^14^ are formed in the temperature range between 0 and about 80 °C [*e.g.* Al_77_R_20_ (ref. 15) and Ga_84_R_20_, R = N(SiMe_3_)_2_ (ref. 16)]. On this basis, metalloid Mg_*n*_X_*m*_/Mg_*n*_R_*m*_ clusters might have been anticipated from metastable MgX or MgR solutions. However, since MgX radicals in metastable solutions rapidly disproportionate above –40 °C to form a mirror of Mg metal and their dihalides, the generation of metalloid magnesium clusters from these highly reactive species seemed for a long while not to be possible. Nevertheless, given our extensive experience with the generation of about 50 metalloid Al/Ga clusters during the last 25 years,^17^ metalloid magnesium clusters remained a sought-after goal. For years, we dreamed of introducing a novel dimension to the chemistry of magnesium in analogy to the new area of aluminum and gallium organometallic chemistry.^12^

In addition to the intermediate character of metalloid clusters, the unprecedented properties of these clusters [*e.g.* superconductivity of the Ga_84_R_20_ cluster^18,19^], and the fundamental interest in the so far unknown arrangement of many Mg–Mg bonds in a molecular species (*cf.* ESI†), there is a further reason for making these Mg-rich clusters a highly exciting subject. With the help of metalloid magnesium clusters one of the mysteries of organometallic chemistry may be solved, *i.e.*, the key to the mechanism of the formation of GRs,^20^ these having already been prepared more than 100 years ago.^21^ Since that time the importance of GR in synthetic chemistry and even in technical chemistry (tens of thousands of tons of industrial chemicals per year^22^) continues to grow as have the exciting development of new applications of GR in synthetic organic chemistry.^23,24^ Over the past 80 years, generations of chemists have made great efforts in investigating the mechanism for the formation of GRs. In particular, the transition from solid magnesium to normal valence RMgX species was a key problem. For a while, it seemed to have been solved, and even today, the formation of Mg(i) halide species is presented in textbooks as plausible intermediates.^25^ However, here we will present the first experimental hint that this formation mechanism is much more complex and that metalloid magnesium clusters play a central role in understanding this important reaction in organometallic chemistry.

## Results and discussion

What are the experimental findings in this novel field of low-valent magnesium chemistry?

The first essential step in this area was the generation of Mg(i) halides: MgX molecules are prepared at about 1000 °C, then trapped at –200 °C (*ca.* 2% of amine in toluene) and finally stored as a metastable solution below –40 °C. This procedure has already been described and improved in our previous work.^11^

Secondly, the redox chemistry of the metastable MgX solution was investigated. MgX species are even stronger reducing agents than isoelectronic Na atoms (*e.g.* for the reduction of diazabutadienes^26^).

After these two tasks were completed, we were prepared to tackle the main challenge, *i.e.*, the generation of the first known metalloid magnesium cluster and the elucidation of its relationship to the formation process of GR. This is described below:

In principle, these clusters are intermediates during the fast disproportionation process of the monohalides, *e.g.* 2 MgCl_(solution)_ → Mg_(solid)_ + MgCl_2(solution)_. In order to trap metalloid clusters as metastable intermediates during this process, the halide radicals have to be tamed, *i.e.* they have to be made slightly more stable *via* substitution of more or less bulky ligands, *e.g.* Cp*. This method has successfully been applied in the generation of the metalloid cluster Al_50_Cp*12.^27^ Nevertheless, the formation of metalloid Al clusters *via* halide substitution and disproportionation of AlX to Al metal and AlX_3_ cannot be easily transferred to the formation of metalloid magnesium clusters. Metastable MgX solutions readily decompose above –40 °C. Therefore, all attempts to perform a simple substitution of, for example, MgBr to MgCp radicals, and then to the more stable (about 200 kJ mol^–1^)^28^ CpMg-MgCp molecule, failed because the disproportionation of MgBr to Mg metal and MgBr_2_ is much faster than the substitution reaction.

The usual methods to accelerate the substitution, *e.g.*, such as increasing the temperature or the addition of ether-like solvents in the case of Al halides, are not possible in the case of MgX chemistry. Any increase of the temperature above –40 °C and any addition of ether into the metastable MgBr solution immediately results in the precipitation of Mg metal, and the dihalide is formed. Consequently, MgX species cannot be formed under the conditions that obtain during the formation of GRs, where usually ether-like solvents are necessary.

Next, we turned to the use of the more reactive KCp*, making a suspension of KCp* and MgBr in toluene and holding it at about –40 °C for several hours to encourage reaction with MgBr. After warming to RT overnight, the solvents were evaporated. The black residue from the disproportionation of MgBr and its reaction with KCp* burns in air. Unexpectedly, it could be partially dissolved in THF. However, all attempts to get a crystalline material from this solution failed. We also checked its many very tiny crystalline-looking particles *via* diffraction experiments (using synchrotron radiation), but without success. Then, we next investigated the solution by mass spectrometry, using an ESI source and the very high resolution of our FT-ICR instrument.

**Fig. 1:**
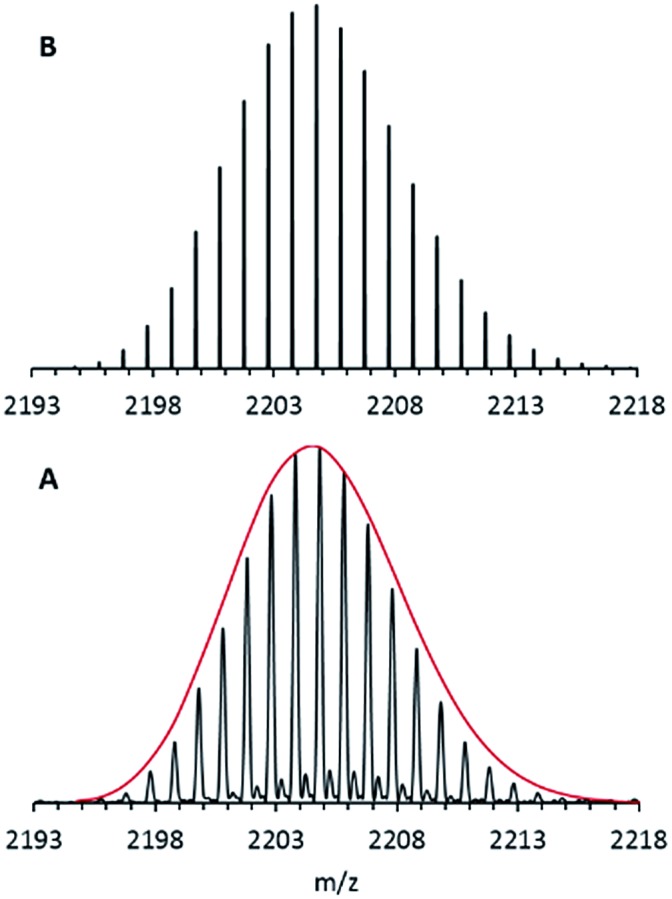
FT-ICR mass spectrum of **1** *via* the ESI method from THF solution: A – experimental data; B – simulation. The red line represents the shape of the simulated isotopic pattern of **1**.

Nevertheless, after using a procedure which had been successfully applied during our electrospray ionization (ESI) experiments with the metalloid cluster anion Ga_19_R_6_ (R = C(SiMe_3_)_3_),^29^ only oxygen-containing magnesium species were obtained. Finally, however, after making some extensive improvements, designed in order to protect all essential parts of the ESI inlet system from air by adding an outside Ar atmosphere, we got a nice isotopically resolved spectrum. Its highest intensity *m*/*z* signal at 2204.80 is shown in Fig. 1.

After calibration of the spectrometer both before and after experiments, the signal of this anion was found to be in accordance with only one formula, *i.e.*, Mg_16_C_102_N_3_Br_4_O_1_K_1_H_173_. Since only the educts: Mg, HBr, KCp*, THF and the amine, NEt_3_ are involved in this reaction, this formula resolves itself into the stoichiometry, Mg_16_Cp*8(NEt_3_)_3_Br_4_(THF)K. A suspension of KCp* and MgBr in toluene turned out to be the “trick” for making this metalloid cluster.

**Fig. 2:**
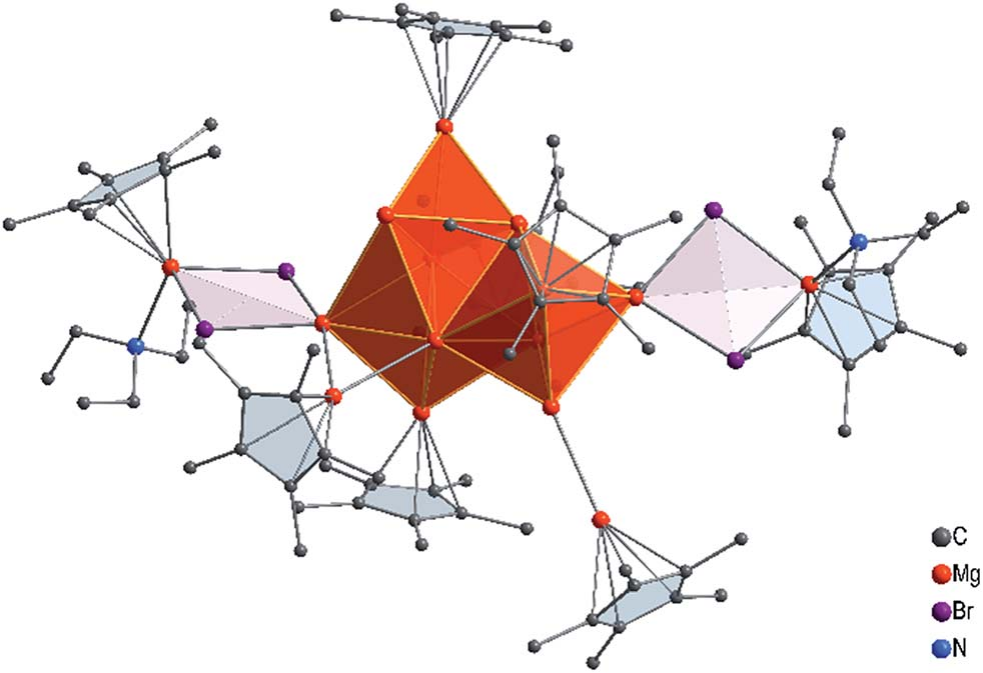
Calculated molecular structure of **1**, the [K(NEt_3_)(thf)]^+^ moiety is omitted for clarity.

With this experimental result in hand, we then turned to computing its structure. First, we performed a number of DFT calculations with structural fragments known from various metalloid Al and Ga clusters (*cf.* ESI†). However, none of the molecules with these structures exhibited a minimum for an [Mg_16_Cp*8(NEt_3_)_3_Br_4_(thf)K]^–^ (**1**) anion. Ultimately, however, a completely different topology for this Mg_16_ cluster showed an energetic minimum, and it is presented in Fig. 2.

**Fig. 3:**
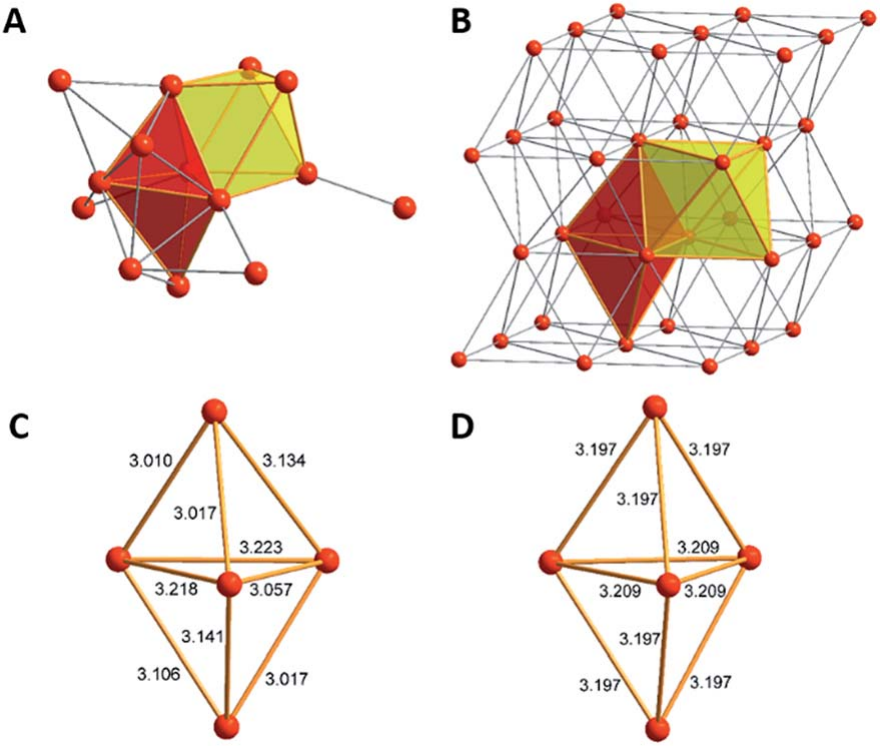
The similar topology of the Mg atoms within Mg_14_ moiety in **1** (A) and the bulk metal (B) is shown. Furthermore, there are similar Mg–Mg distances in **1** (C) and in the bulk metal (D).

The core of this Mg_16_ cluster **1**, presented in Fig. 3, contains 14 Mg atoms in an arrangement which is very similar to that of the structure of hcp Mg metal, *i.e.*, containing a connected octahedron with a trigonal bipyramid. Thus, in retrospect, it is understandable that none of the structures of metalloid Al/Ga clusters exhibited a similar topology to that of **1**, because the atoms in solid Al and in the high pressure allotrope of Ga are fcc packed. In order to show the similarity between the Mg–Mg distances in **1** and those of bulk Mg, the distances within the trigonal bipyramid fragment are shown in Fig. 3.

How many direct Mg–Mg contacts/bonds make **1** into a real metalloid cluster? There are 5 Mg–Mg bonds containing an additional bromine atom (Mg–MgBr), 13 Mg-MgCp* units and 9 bonds between “naked” Mg atoms; thus there are altogether 27 Mg–Mg bonds!! Furthermore, cluster **1** has two additional Mg atoms with the oxidation number +2 within the GR moiety, Cp*MgBr. For the Mg_14_ core without the GR moieties the oxidation number is only 0.43.

What about the energetic situation of **1** with respect to:

(a) its generation from MgBr, as observed experimentally and

(b) the formation of the GR?

**Fig. 4:**
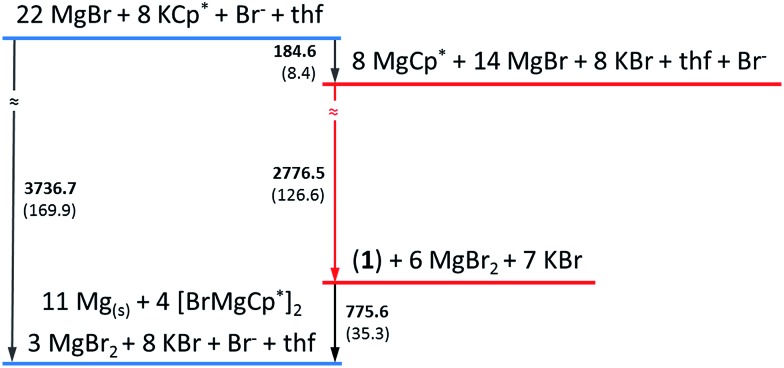
The calculated simplified energetic ladder (energy: kJ mol^–1^; in brackets values calculated per one mole of Mg atom) for the formation of GR *via* the metalloid cluster **1** as an intermediate step. Detailed information are given in Fig. S1 in ESI.† In contrast, the [Mg(NEt_3_)Br] radical is only an assumed intermediate (*cf.* text), which, for example, would immediately decompose in ether-like solution to solid Mg and MgBr_2_.

In a simplified calculated thermodynamic diagram (Fig. 4) MgBr molecules are partially substituted first by Cp*, *i.e.*, towards MgCp* radicals. However, Mg monohalide and MgCp* radicals have never been observed during the formation of GR. This missing observation of MgX and MgR now seems plausible based on:

(a) our experiments, showing that MgBr/MgCp* species would immediately decompose to Mg metal and MgBr_2_/MgCp*2 above –40 °C and even at lower temperature in the presence of ether-like solvents;

(b) by their highly exothermic and consequentially fast reaction to the soluble (in ether) and temperature stable (at room temperature) metalloid cluster **1** and also to the observed products Mg_(solid)_, MgBr_2_ and GR.

Therefore, metalloid clusters, such as **1** and not the MgBr radical are possibly intermediates during the formation of the Grignard reagent, Cp*MgBr. This conclusion is also impressively confirmed *via* the calculated exothermic reaction of Mg_(solid)_ with BrCp* to form **1** in contrast with the endothermic reaction to MgBr and Cp* radicals (*cf.* ESI†).

## Conclusions

The results presented here show for the first time:

(1) that, like the many so far singular and unprecedented examples of well investigated metalloid Al_*n*_R_*m*_/Ga_*n*_R_*m*_ clusters,^13^ it is also possible to generate Mg_*n*_R_*m*_ clusters; although it requires somewhat more experimental effort, and

(2) a molecular cluster with many Mg–Mg bonds, *i.e.*, **1**, in contrast to the previously reported molecular examples, each having only one Mg–Mg single bond;

(3) that it is not the highly reactive MgBr radical, which spontaneously forms Mg metal and MgBr_2_ with THF, but instead the metalloid Mg_16_ cluster, **1**, which is stabilized by the coordination of already two BrMgCp* Grignard entities and which is soluble in THF, that is an intermediate on the way from Mg metal to the formation of the GR Cp*MgBr.

Thus, after about 80 years of intensive investigation concerning the mechanism of the GR formation process,^30^ the Mg_16_ metalloid cluster provides a new pathway for understanding it. Not only are the above discussed oxidation numbers, *i.e.*, 0, 1 and 2, of magnesium involved, but there are also intermediate oxidation states between 0 and +2 present within metalloid Mg clusters as well, all of these testifying to the complexity of a seemingly simple process.

To summarize, the Mg_16_ cluster (**1**) like every metalloid cluster, defined as clusters containing more metal–metal than metal ligand bonds, reflects with its 14 central Mg atoms the topology of Mg atoms in the Mg metal itself. Though metalloid clusters are essential building blocks for the basic understanding of fundamental processes such as the dissolution/formation of metals, and though in the cases of Al_*n*_R_*m*_ and Ga_*n*_R_*m*_ (*n* > *m*) metalloid clusters, they have been under discussion for nearly two decades, they seem not to be known or, at best, to be known only as a curiosity for the majority of chemists. In contrast, nanoscaled metalloid gold clusters like the remarkable Au_130_R_50_ (ref. 31) are recently highlighted. Nevertheless, as intermediates between the solid metal and its usually oxidized compounds, these types of molecular clusters start to solve one of the mysteries of organometallic chemistry, *i.e.*, the mechanism of the formation of GR, which should be reevaluated as a highly complex process rather than a seemingly simple one. Therefore, in the near future, metalloid clusters of Mg, like those of other metals, may exist as intermediates during their manifold heterogeneous reactions, and these may enter into textbooks as the basis of fundamental knowledge. Thus, **1** may open a new area of research, *i.e.*, the role of metalloid (metal rich) clusters in fundamental chemical processes (*e.g.* chemical and electrolytic dissolution of bulk metals) and their investigations *via* spectroscopy, theory and synthetic chemistry.
