# Supplementary material for: Many Mg–Mg bonds form the core of the Mg16Cp*8Br4K cluster anion: the key to a reassessment of the Grignard reagent (GR) formation process?
Source: Chem Sci. 2015 Nov 26;7(2):1543–7. doi: 10.1039/c5sc03914b (PMC5530812; doi:10.1039/c5sc03914b)
Supplement: Supplementary file 1 [file SC-007-C5SC03914B-s001.pdf]

Electronic Supplementary Material (ESI) for Chemical Science.
This journal is © The Royal Society of Chemistry 2015

Electronic supplementary information (ESI)

Many Mg-Mg bonds form the core of the $Mg_{16}Cp^*_8Br_4K$ cluster anion: The key to a reassessment of the Grignard Reagent (GR) formation process?

T. Kruczyński, F. Henke, M. Neumaier, K. H. Bowen, H. Schnöckel

correspondence to: hansgeorg.schnoeckel@kit.edu

This PDF file includes:

1. **Materials and Methods** 2

1.1. Experimental 2
1.2. MS investigation 2

2. **Supplementary Text** 6

2.1. Metalloid clusters 6
2.2. Mg_x species 6
2.3. $[Mg_{28}Cp^*_8Br_3(NEt_3)_2]^-$ (2) cluster 6

3. **References and Notes** 9

Other Supplementary Materials for this manuscript includes the following:

Additional Data table S3: XYZ coordinates of the compound **1**.

1. Materials and Methods

1.1. Experimental

The experiments were carried out in an atmosphere of dry argon (99.998 %) using typical Schlenk technique. Toluene and THF were dried over potassium/benzophenone. NEt_3 was dried over potassium and afterwards distilled prior to use. The metastable magnesium monobromide solution was obtained in the co-condensation experiment according to the earlier published procedure.[1] In this process 100 cm^{-3} of toluene was cocondenced together with 2 cm^{-3} of NEt_3 and 20 mmol of MgBr resulting in deep brown homogenous solution (c_{Br} = 0.0523 mol·dm^{-3}).
0.555 g of KCp^* was reacted with 50 cm^{-3} of the metastable MgBr solution. The reaction was performed at about -70°C. After 2 hours of stirring the reaction mixture was slowly warmed to room temperature overnight. A black powder together with clear brown solution was formed. After few days at room temperature all the volatile substances were removed under reduced pressure. The solid residue was extracted with a few cm^{-3} of THF. This extract was investigated using ESI-MS methods.

1.2. MS investigation

Mass spectra were recorded on an IonSpec Ultima FT-ICR-MS (Fourier-Transform Ion Cyclotron Resonance Mass Spectrometer), equipped with a 7 T, actively shielded, superconducting magnet. The instrument was coupled to a modified electrospray ionization (ESI) source (Analytica of Branford). To protect highly air-sensitive clusters from oxidation during the electrospray process the home-built chamber of the ESI source can be evacuated (to 10^{-1} mbar) and flushed with argon (Air Liquide / 99.999%) as an inert gas. The cluster anion **1** was brought into the gas phase by electrospraying its solution in THF and nitrogen (Air Liquide / 99,999%) was used as a nebulizing gas. The electrospray needle was held at ground potential and the potentials of the endplate and the entrance of the nickel coated fused silica desolvation capillary were held at 3.4 kV and 3.6 kV, respectively.

In order to improve the signal-to-noise ratio the ions were pre-trapped and accumulated in a hexapole ion trap for 10 s before they were transferred into the ICR cell via a quadrupole ion guide and by increasing the potential of the entrance trapping electrode from -20 V to 0 V. After capturing the ions both trapping electrode potentials were set to -0.5 V and ion detection took place by standard ICR techniques. The transient signal was typically recorded for 524 ms (2048 K data points) yielding the mass spectra after Fourier transformation. After calibrating the signal of **1** with spectra of anionic cesium iodide clusters taken under the same experimental conditions, we determined the mass of the most intense signal of the isotopic distribution to m/z = 2204.80 ± 0.05.

1.3. DFT and thermodynamic considerations

Theoretical investigations based on DFT methods were performed using the Turbomole software package (version 6.3, 6.4 or 6.5) [2]. The geometries of all molecules were optimised using def-SV(P) basis sets.[3] In calculations, Coulomb interactions were treated within the resolution of the identity (RI)[4] approximation together with Becke-Perdew-86 functional.[5, 6] The grid required for the numerical integration of exchange and correlation contributions was of the m4 size. The energetic minimum of the calculated geometries was

confirmed by the vibrational analyses performed analytically (aoforce) or numerical (NumForce).[7, 8] Molecules in an energetic minimum have no negative eigenvalues.
The calculations were performed at the Steinbuch Centre for Computing, KIT Karlsruhe, on a parallel computer HP XC3000 applying for each calculation 1 to 8 nodes (each equipped with two Quad-core Intel Xeon processors E5540 Nehalem, 2.53 GHz) together with 3 to 6 GB operating memory per core per calculation.

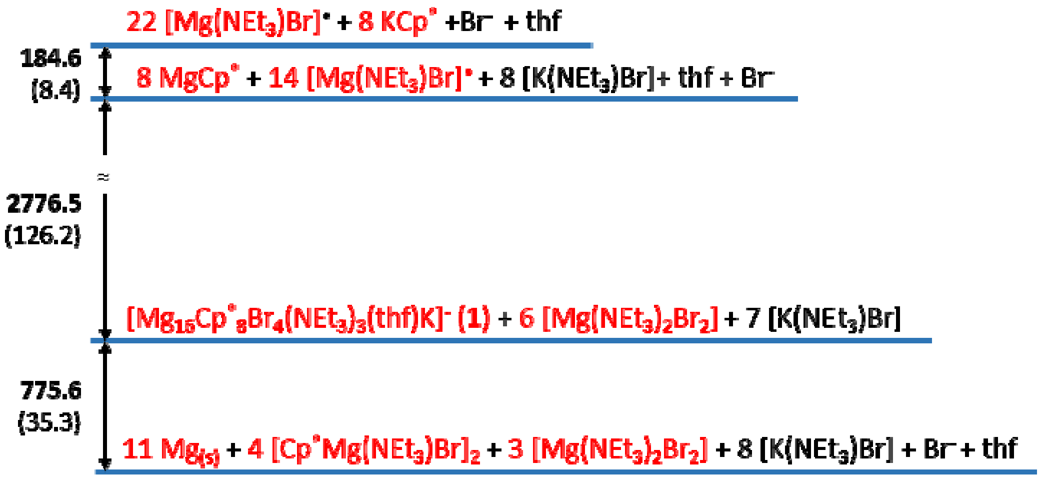

Fig. S1 Detailed diagram presented in **Fig. 4** (energy: kJ·mol^{-1}, in brackets values calculated per one mole of Mg atoms).

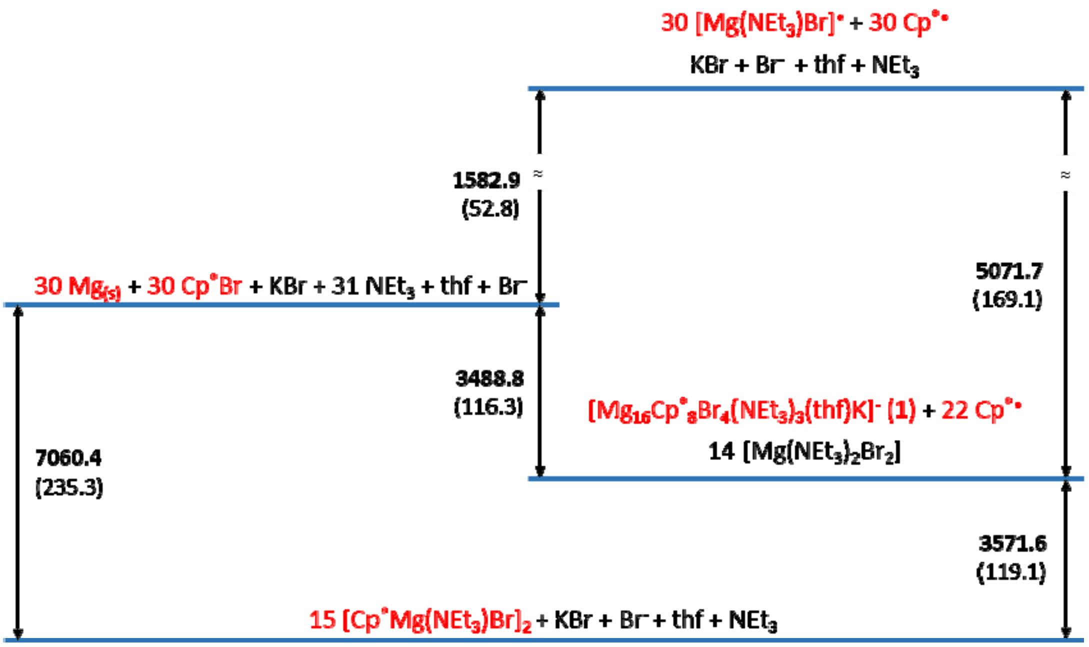

Fig. S2 The calculated thermodynamic ladder (energy: kJ·mol^{-1}, in brackets values calculated per one mole of Mg atoms) for the formation of GR via the metalloid cluster **1** and the [Mg(NEt_3)Br] radical.

Table S1 Calculated energy and other parameters.

Compound	Symmetry	Energy [kJ·mol^{-1}]	HOMO-LUMO gap [eV]
$MgCp^*_2$	C_2	-2572108.5115	3.79
$MgCp^*$	C_1	-1548556.2822	1.31
THF	C_1	-609826.6989	6.31
NEt_3	C_1	-767098.5290	5.72
MgBr	C_{6v}	-7283642.7202	1.18
Br^-	C_1	-6758530.7730	
KBr	C_{6v}	-8333611.6507	2.08
Mg	C_1	-525147.3451	
$[Cp^*Mg(NEt_3)(\mu\text{-}Br)]_2$	C_1	-18148805.0192	3.75
$[Mg_{16}Cp^*_8Br_4(NEt_3)_3(thf)K]^-$ (**1**)	C_1	-48111826.2035	0.99
$[Mg(NEt_3)Br]$	C_1	-8050815.9829	1.21
$[Mg(NEt_3)_2Br_2]$	C_1	-15576655.7126	5.18
Cp^*Br	C_1	-7781774.1888	2.64
$[Mg_{28}Cp^*_8Br_3(Et_3N)_2]^-$ (**2**)	C_1	-44703609.9656	0.89
$Cp^{*\bullet}$	C_1	-1023298.4153	0.82
$K(NEt_3)Br$	C_1	-9100752.4789	2.72

2. Supplementary Text

2.1. Metalloid clusters

from [9]:

“We have described such clusters, which contain both ligand-bearing and naked metal atoms that are only bonded to other metal atoms, as metalloid, to express, in accordance with the Greek word *ειδος* (ideal, prototype), that the ideal form or the motif of the solid structure can be recognized in the topology of the metal atoms in the cluster. The original limits of the term metalloid used, for example, for the elements silicon and germanium which are metal-like with respect to certain macroscopic properties (e.g. metallic luster) were extended to include the metalloid clusters, thus accessing an additional structural level, which was only possible through crystal structure analysis. In general such metalloid clusters contain more direct metal-metal contacts than metal-ligand contacts. This means that metalloid clusters represent a subgroup of the extensive metal-atom cluster group in which, according to the definition of *Cotton*,[10] non-metal atoms may also be present.”

2.2. Mg_x species

There are some reports about reactions of Mg_x species (x = 1, 2, 3, 4, n) from metal vapor deposition (MVD) procedure with RX (R = alkyl, aryl; X = halide) molecules. The products are investigated via matrix spectroscopy[11, 12] and via matrix assisted laser desorption/ionization mass spectroscopy (MALDI-MS).[13] In the latter case, $[RMg_4X+H]^+$ cations are formed during heating the sensitive samples by the laser desorption process.

2.3. $[Mg_{28}Cp^*_8Br_3(NEt_3)_2]^-$ (2) cluster

During the preliminary analysis of the mass spectroscopic results the most intensive signal of the spectrum was assign to the $[Mg_{28}Cp^*_8Br_3(NEt_3)_2]^-$ (**2**) cluster ion. In-depth investigation excluded this species as being responsible for the observed signal. Nevertheless, structure of the cluster **2** was calculated because this cluster has a similar molecular mass to **1** and provides addition information about possible topology of the metalloid magnesium clusters. Moreover, calculated thermodynamic data are in line with the one obtained for **1** and previously reported values.[14]

Calculated molecular structure of **2** is presented in Fig. S3.

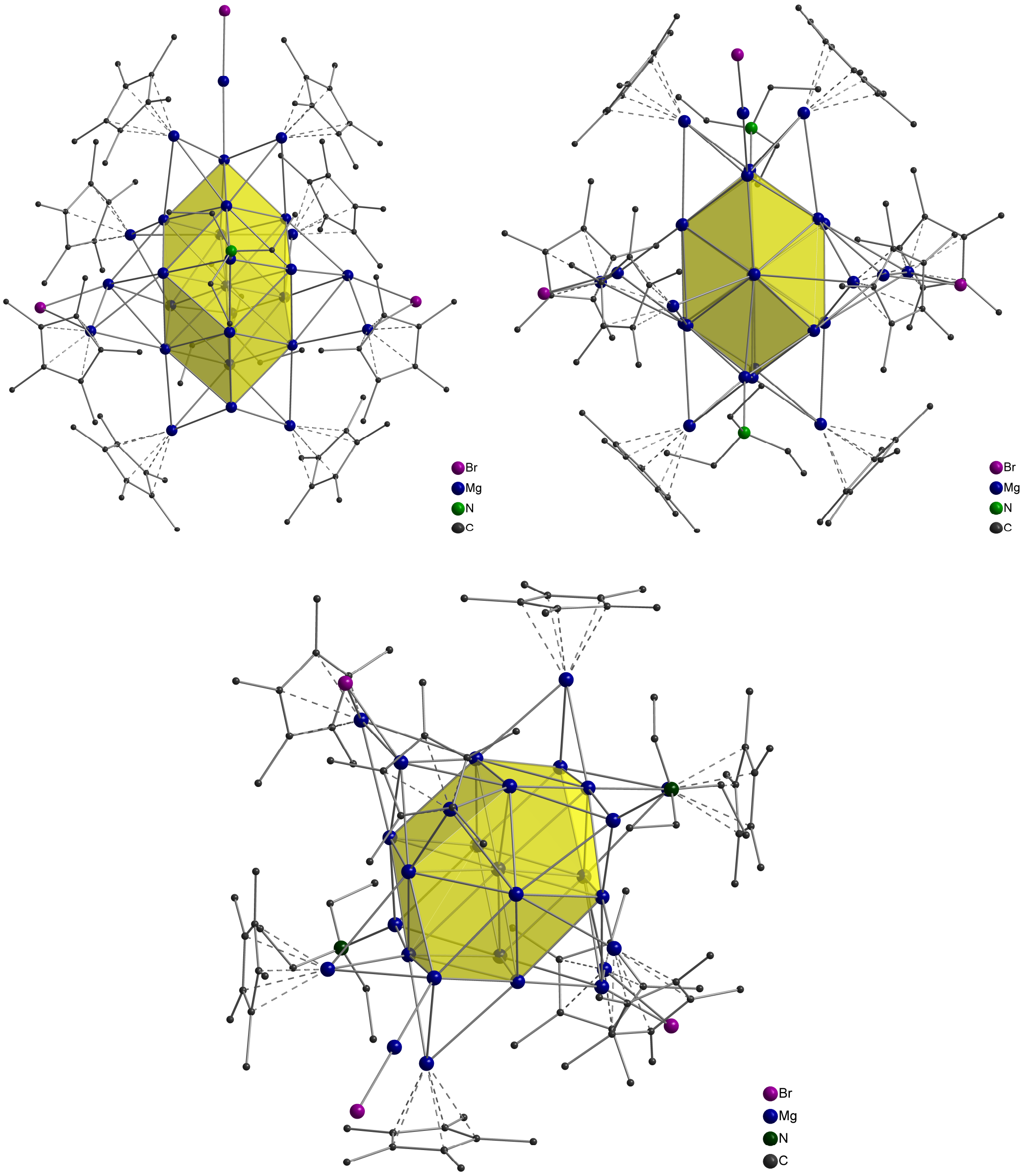

Fig. S3 The calculated molecular structure of **2**.

The cluster **2** contains 28 magnesium atoms which form metallic core of the molecule in which *fcc* close-packing of Mg atoms is visible. This arrangement is compared with the structure of bulk magnesium (Fig. S4).

Based on the analysis of the electron density of the icosahedral Mg_{13} units found within cluster **2** and bulk Mg metal, volumes of both metal fragments were compared in table **2**. Details of similar calculations can be found in the reference.[15]

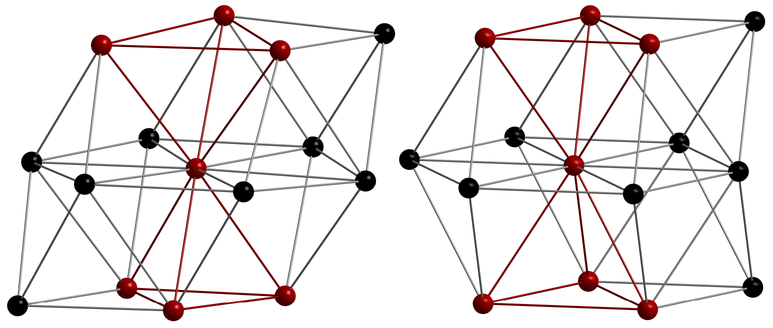

Fig. S4 Arrangement of the Mg atoms in the structure of **2** (left) and bulk metal (right).

Table S2 Volume of Mg_{13} unit found in the structure of solid Mg and structure of **2**.

**Structure**	**Volume** [Å^3]
$Mg_{(s)}$	559.45
**2**	499.01

3. References and Notes

1. T. Kruczyński, N. Pushkarevsky, P. Henke, R. Köppe, E. Baum, S. Konchenko, J. Pikies and H. Schnöckel, *Angew. Chem., Int. Ed.*, 2012, **51**, 9025-9029.
2. R. Ahlrichs, M. Bär, M. Häser, H. Horn and C. Kölmel, *Chem. Phys. Lett.*, 1989, **162**, 165-169.
3. A. Schäfer, H. Horn and R. Ahlrichs, *J. Chem. Phys.*, 1992, **97**, 2571-2577.
4. K. Eichkorn, F. Weigend, O. Treutler and R. Ahlrichs, *Theor Chem Acta*, 1997, **97**, 119-124.
5. A. D. Becke, *Phys. Rev. A*, 1988, **38**, 3098-3100.
6. J. P. Perdew, *Phys. Rev. B*, 1986, **33**, 8822-8824.
7. O. Treutler and R. Ahlrichs, *J. Chem. Phys.*, 1995, **102**, 346-354.
8. P. Deglmann, F. Furche and R. Ahlrichs, *Chem. Phys. Lett.*, 2002, **362**, 511-518.
9. A. Schnepf and H. Schnöckel, *Angew. Chem., Int. Ed.*, 2002, **41**, 4170-4170.
10. F. A. Cotton, *Quarterly Reviews, Chemical Society*, 1966, **20**, 389-401.
11. Y. Imizu and K. J. Klabunde, *Inorg. Chem.*, 1984, **23**, 3602-3605.
12. K. J. Klabunde and A. Whetten, *J. Am. Chem. Soc.*, 1986, **108**, 6529-6534.
13. L. A. Tjurina, V. V. Smirnov, G. B. Barkovskii, E. N. Nikolaev, S. E. Esipov and I. P. Beletskaya, *Organometallics*, 2001, **20**, 2449-2450.
14. T. Holm, *J. Organomet. Chem.*, 1973, **56**, 87-93.
15. H. Schnöckel and A. Schnepf, in *The Group 13 Metals Aluminium, Gallium, Indium and Thallium: Chemical Patterns and Peculiarities*, John Wiley & Sons, Ltd, 2011, DOI: 10.1002/9780470976548.ch7, pp. 402-487.

Table S3

XYZ coordinates of the compound **1**.

Element	X	Y	Z
C	7.9649022	0.0334139	3.0535594
C	8.9451044	-1.0014763	2.8637775
C	8.2852468	-2.2730292	3.0242446
C	6.9014073	-2.0188856	3.3255096
C	6.701989	-0.5935458	3.3442717
C	10.4328405	-0.7636322	2.7708053
C	8.9423084	-3.6292204	3.1076674
C	5.8832087	-3.062117	3.7062729
C	5.4225344	0.1219257	3.6918588
C	8.241975	1.5146834	3.0886297
Mg	7.1309298	-1.1444981	1.063275
Br	4.7541948	-2.198653	0.349309
Br	6.2199911	1.128382	-0.0389839
N	8.4687766	-1.9196882	-0.7442441
Mg	3.6691152	0.21917	-0.2339666
Mg	0.8508777	-0.7119103	-1.0145676
Mg	-0.2964815	2.293924	-1.0852711
Mg	-1.8771451	4.3141245	-2.509383
C	-2.098139	4.9876084	-4.8186404
C	-1.2957925	5.9935083	-4.1731088
C	-2.1214135	6.6617855	-3.2014864
C	-3.4321685	6.0655524	-3.2435917
C	-3.4157555	5.026853	-4.2402237
C	0.1295897	6.352891	-4.5081476
C	-1.6794613	4.1168615	-5.97468
C	-4.6001553	4.2084992	-4.6882543
C	-4.650104	6.5545135	-2.5022695
C	-1.7337425	7.8880897	-2.4142888
Mg	-1.8569058	-0.0426983	-2.1643428
Mg	-1.4693292	-2.6213466	-0.6368403
C	-0.7417275	-5.0321127	-0.2354765
C	-0.6327115	-4.8815807	-1.6646266
C	-1.9516691	-4.6619908	-2.1937953
C	-2.879797	-4.6670157	-1.0910466
C	-2.1345356	-4.90988	0.1217374
C	-4.3748429	-4.4859433	-1.1978516
C	-2.3127638	-4.3927908	-3.6351841

C	0.6652917	-4.9167979	-2.4336084
C	0.4272048	-5.2815122	0.6875027
C	-2.7350132	-5.0552206	1.4996465
Mg	2.3052636	1.0423839	-2.9894914
C	3.9968673	-0.0651665	-4.4271325
C	4.118474	1.3535072	-4.6212024
C	2.891932	1.8289214	-5.2030447
C	2.0082809	0.7002779	-5.3627296
C	2.6969656	-0.4697141	-4.8846066
C	0.6493458	0.706846	-6.0140093
C	2.6327226	3.2371577	-5.6708613
C	5.3428289	2.1840394	-4.3316622
C	5.0707502	-0.9718654	-3.8864638
C	2.1654339	-1.8782449	-4.9261243
Mg	2.5781607	3.1768446	-0.7518852
Mg	4.3254222	5.4473065	-0.6734741
C	4.8315248	7.7929056	-1.0780793
C	5.096816	7.5319444	0.3129101
C	6.1811042	6.5879479	0.3886269
C	6.5885793	6.2685042	-0.9542377
C	5.7541899	7.0134094	-1.8613928
C	7.7393402	5.3689053	-1.3307128
C	6.8325386	6.0758027	1.6487299
C	4.4172983	8.198732	1.482794
C	3.8252275	8.7778928	-1.6183556
C	5.8787301	7.0432507	-3.3643694
Mg	-1.3852164	0.0294405	0.9332567
Mg	-3.1504996	2.2266958	-0.1583608
Br	-4.7783085	0.8142599	1.7478982
Br	-5.5371947	3.0817899	-0.9807471
Mg	-7.0712733	1.4929819	0.6022583
C	-9.3293864	0.7714501	-0.3542499
C	-9.0062151	-0.173181	0.68532
C	-7.7833682	-0.8351085	0.317052
C	-7.3519284	-0.3059162	-0.9497351
C	-8.3062344	0.6897594	-1.3618063
C	-9.8901947	-0.5800906	1.8395481
C	-10.6219916	1.5342391	-0.511956
C	-8.3225159	1.4067916	-2.6873277
C	-6.1575702	-0.7706443	-1.7408489
C	-7.1363115	-1.974195	1.0622423
N	-7.8040014	3.0838621	2.2030537

Mg	1.3251844	1.2836022	1.3639825
Mg	-0.2792247	0.2943349	3.7359954
C	0.6751715	-1.0634651	5.5187213
C	-0.0858556	-0.0146991	6.1510839
C	-1.4722015	-0.2053213	5.8078086
C	-1.5649554	-1.3704127	4.9682927
C	-0.2413601	-1.9036082	4.797176
C	-2.8421963	-1.969525	4.4384148
C	-2.652343	0.5787328	6.3243384
C	0.4736986	0.97934	7.136861
C	2.1551264	-1.2878198	5.6962025
C	0.1090444	-3.1325557	3.9983755
Mg	-0.9405789	3.3372146	1.7751375
C	0.221063	5.1427111	2.9520953
C	-0.4928924	5.7579946	1.8674327
C	-1.9003297	5.5773518	2.0991904
C	-2.0585573	4.8447244	3.3273794
C	-0.7473472	4.5781602	3.8538288
C	-3.3555934	4.4793845	4.0009143
C	-2.9987904	6.1295399	1.2301138
C	0.1102427	6.5182892	0.7161639
C	1.7134601	5.1515565	3.1591526
C	-0.4516269	3.9172328	5.1716749
H	-2.7947442	5.9788999	0.1445809
H	-3.1406206	7.2283882	1.3787204
H	-3.9780721	5.644235	1.4366618
H	0.5603108	3.4551681	5.1883869
H	-1.1808815	3.1085004	5.4013116
H	-0.489618	4.6386585	6.025777
H	-4.2136619	4.5463421	3.2972852
H	-3.5885631	5.1535874	4.8619768
H	-3.3453945	3.4389756	4.3989063
H	1.1757243	6.2442971	0.5477382
H	0.0811449	7.6236976	0.8800402
H	-0.4243499	6.3241372	-0.2416956
H	2.2705779	5.2029407	2.1966079
H	2.067272	4.2359231	3.6849255
H	2.0468554	6.0255872	3.7710291
H	3.0531058	3.998565	-4.9766427
H	1.5445343	3.4451744	-5.7592924
H	3.0814073	3.4328659	-6.6756394
H	2.6509418	-2.5247212	-4.1614842

H	2.3357545	-2.3636784	-5.9194595
H	1.0701112	-1.9161833	-4.7293158
H	-0.0278854	-0.0560984	-5.5678255
H	0.707764	0.488017	-7.1090778
H	0.1382977	1.6877193	-5.9067451
H	5.8666841	1.853674	-3.4065615
H	5.0880899	3.2571716	-4.1878884
H	6.0880926	2.1373781	-5.1639498
H	5.7599959	-1.3379327	-4.6877376
H	4.6448052	-1.8709215	-3.3876877
H	5.7019621	-0.451978	-3.1310673
H	-1.993186	4.5501776	-6.9561671
H	-2.1209548	3.0969325	-5.9182546
H	-0.5760178	3.992164	-6.0181221
H	-2.2480824	7.9412659	-1.4298699
H	-1.9928087	8.8262261	-2.9646586
H	-0.641179	7.927902	-2.2133869
H	0.701208	5.4812027	-4.8935074
H	0.6828032	6.7301123	-3.6196709
H	0.188561	7.1506043	-5.2888352
H	-4.2912328	3.2182204	-5.0909012
H	-5.1756162	4.7204445	-5.4991255
H	-5.3090785	4.0121338	-3.854548
H	-5.3245106	5.7201495	-2.2096681
H	-5.2504631	7.262902	-3.1253855
H	-4.3780025	7.0974953	-1.5707523
H	-2.459315	-3.3021369	-3.831827
H	-3.2586186	-4.9005963	-3.9358474
H	-1.5135382	-4.7306993	-4.3311126
H	-1.9810848	-4.8889606	2.2981575
H	-3.183377	-6.0662769	1.6804913
H	-3.5489185	-4.3170095	1.6751495
H	-4.8061932	-4.053998	-0.2686761
H	-4.9261531	-5.4445621	-1.3867782
H	-4.6469965	-3.7947884	-2.0248629
H	1.2476857	-3.9702095	-2.3184
H	0.4989672	-5.0509875	-3.524973
H	1.3344723	-5.7405017	-2.08893
H	0.1121722	-5.3177695	1.7520628
H	1.2035836	-4.4858596	0.6092676
H	0.9480287	-6.2457862	0.4622302
H	6.1330091	6.0880291	2.5135203

H	7.7183133	6.6912892	1.9429558
H	7.1928728	5.0287322	1.5367633
H	4.9007238	7.2303067	-3.8613871
H	6.2711483	6.0847774	-3.7710106
H	6.5729181	7.8478611	-3.7117459
H	7.8089021	4.476564	-0.6693012
H	8.7217821	5.8986196	-1.2662843
H	7.6452121	4.9911614	-2.3724183
H	3.360257	8.462906	1.2580637
H	4.9300602	9.1469485	1.7789429
H	4.402025	7.5474776	2.384608
H	2.9459454	8.8881416	-0.9457727
H	3.4351968	8.4738283	-2.6154145
H	4.2630714	9.7989343	-1.7441569
H	-2.3502083	1.5652819	6.7376547
H	-3.1835301	0.0348814	7.1439469
H	-3.4061831	0.7724343	5.5277019
H	1.204916	-3.214961	3.8311586
H	-0.3691287	-3.1228273	2.9910495
H	-0.215992	-4.0732557	4.5079351
H	-2.6560129	-2.6057956	3.5458174
H	-3.5757292	-1.1938375	4.1249841
H	-3.3487562	-2.6124391	5.2010789
H	1.4535752	1.3945061	6.8099109
H	0.6441306	0.5119777	8.1380937
H	-0.2078074	1.8420171	7.2952086
H	2.7172524	-0.3306812	5.7694515
H	2.5990801	-1.8549319	4.8489527
H	2.3818103	-1.8649828	6.626505
H	-10.4692496	2.5804809	-0.8642177
H	-11.2008238	1.5869946	0.4359326
H	-11.2925883	1.0501799	-1.2640627
H	-6.026122	-1.9060265	1.0419506
H	-7.4191843	-2.9648959	0.6273601
H	-7.4371493	-1.9945176	2.1329617
H	-9.3390196	-0.6670738	2.8041734
H	-10.3643591	-1.576366	1.6611778
H	-10.7240572	0.1374056	2.0022459
H	-7.2970122	1.6201983	-3.0584181
H	-8.852852	2.3831946	-2.6292295
H	-8.8411022	0.8049378	-3.4740214
H	-5.7589412	0.0307163	-2.4005749

H	-6.4062679	-1.6394653	-2.3991356
H	-5.3184683	-1.0918778	-1.0839173
H	9.980122	-3.6198221	2.7082744
H	8.383294	-4.4181928	2.5527086
H	9.0148507	-3.9864576	4.1644621
H	9.1712832	1.7774941	2.5364433
H	8.373336	1.884449	4.1348598
H	7.4167696	2.1052687	2.6335132
H	10.7094482	0.0008357	2.0083624
H	10.9933204	-1.6921895	2.5256715
H	10.8460454	-0.3948276	3.7413327
H	4.8521417	-2.7569201	3.4266262
H	5.8855919	-3.253517	4.807644
H	6.0760164	-4.0380289	3.2073084
H	5.363034	1.1215979	3.2090495
H	5.3236452	0.283809	4.7927078
H	4.5228912	-0.4475841	3.3699103
C	-7.0515165	2.8303041	3.4737279
C	-9.2787172	2.9839828	2.4093801
C	-7.402864	4.441167	1.7091697
C	8.7356995	-0.7467376	-1.6374132
C	7.6602263	-2.9062953	-1.5303317
C	9.7359669	-2.5231317	-0.2371328
H	9.1859665	-1.1082346	-2.5950349
H	7.7454906	-0.3134704	-1.8908874
C	9.6154037	0.3460781	-1.0325766
H	8.1518693	-3.0796394	-2.519859
C	7.4148683	-4.2470216	-0.8383677
H	6.6830785	-2.4207176	-1.7366913
C	10.7229266	-3.057237	-1.2872942
H	10.2356108	-1.7569525	0.3856929
H	9.4550949	-3.3370193	0.4595639
H	11.6059322	-3.4867266	-0.7642111
H	10.2873586	-3.8639092	-1.9164395
H	11.0979624	-2.2610046	-1.9661656
H	9.6028285	1.2294115	-1.7064958
H	9.2250996	0.6863681	-0.0488047
H	10.674724	0.0328958	-0.9070581
H	6.6615945	-4.8166435	-1.4242002
H	8.3307193	-4.8732869	-0.7656381
H	6.9907825	-4.1084988	0.1798142
H	-7.4823241	5.1768606	2.5473058

H	-6.3263023	4.3730792	1.4429156
C	-8.1859508	4.9539798	0.5009268
H	-7.2216735	3.6826086	4.1768835
C	-7.3753781	1.5107814	4.1720108
H	-5.9736031	2.8401045	3.2083947
C	-9.9042358	3.9359808	3.4408529
H	-9.7576459	3.1384537	1.4233283
H	-9.4994245	1.9349941	2.688171
H	-11.0032047	3.7646814	3.4675372
H	-9.5194228	3.7666586	4.4698176
H	-9.7435449	5.0067586	3.1887196
H	-6.6538735	1.3624732	5.0040079
H	-8.3994141	1.4841896	4.6042862
H	-7.2525828	0.6464729	3.4833628
H	-7.6792765	5.8599741	0.1051259
H	-8.200625	4.2096094	-0.3245221
H	-9.2327238	5.2338267	0.750022
K	-1.9871635	-7.5460625	-1.284404
N	-3.9421204	-9.316172	-2.6461194
O	-0.4020575	-9.207158	0.2344708
C	-0.4672496	-9.1002607	1.6816652
C	0.8756157	-9.7595505	-0.1589449
C	1.7759399	-9.6462176	1.0754521
C	0.7630257	-9.8436593	2.2176869
H	1.2529233	-9.1867624	-1.0344568
H	0.7365695	-10.823707	-0.4687265
H	-1.4304981	-9.5384767	2.0243372
H	-0.4481742	-8.0214225	1.9607869
H	1.1132961	-9.4364706	3.1890528
H	0.5392451	-10.9246474	2.3579528
H	2.2321687	-8.6335122	1.1305109
H	2.5958439	-10.3949621	1.0782834
C	-5.1311622	-8.9667379	-1.8336845
H	-5.2574134	-7.8614317	-1.9019542
H	-4.8936168	-9.1803626	-0.7669208
C	-6.4658193	-9.6436949	-2.1939102
C	-4.0812212	-8.8754639	-4.0523092
C	-2.7493322	-8.6329794	-4.7737415
H	-4.6572756	-7.9237926	-4.0497023
H	-4.6903169	-9.6078338	-4.6444929
C	-3.574164	-10.7446136	-2.5741847
C	-3.214697	-11.2524396	-1.1738252

H	-2.6927814	-10.888503	-3.236235
H	-4.3841848	-11.3916447	-3.0058225
H	-2.1838454	-7.7846361	-4.3264365
H	-2.9401921	-8.3655692	-5.835883
H	-2.0921504	-9.5303373	-4.7771081
H	-2.3846116	-10.6673046	-0.7158314
H	-2.8775988	-12.309954	-1.2421816
H	-4.0800887	-11.2350228	-0.4761632
H	-6.7714044	-9.4289452	-3.2411403
H	-7.2691174	-9.2588372	-1.5281861
H	-6.4290708	-10.7476928	-2.0659618
